# The Dark Side of Relational Leadership: Positive and Negative Reciprocity as Fundamental Drivers of Follower’s Intended Pro-leader and Pro-self Unethical Behavior

**DOI:** 10.3389/fpsyg.2020.01473

**Published:** 2020-07-10

**Authors:** Tim Vriend, Ramzi Said, Onne Janssen, Jennifer Jordan

**Affiliations:** ^1^Department of Human Resource Management and Organizational Behavior, University of Groningen, Groningen, Netherlands; ^2^IMD, Lausanne, Switzerland

**Keywords:** leader–member exchange, positive reciprocity, negative reciprocity, social exchange theory, pro-leader unethical intention, pro-self unethical intention

## Abstract

In this study, we use a social exchange perspective to examine when [i.e., high- vs. low-quality leader–member exchange (LMX)], why (i.e., positive vs. negative reciprocity), and how (i.e., pro-leader vs. pro-self unethical behavior) followers consider unethical behavior that either benefits the leader or the self. Across an experimental and a time-split survey study, we find that high-quality LMX relationships motivate pro-leader unethical intention as a means to satisfy positive reciprocity motives, and that low-quality LMX relationships motivate pro-self unethical intention as a means to satisfy negative reciprocity motives. Importantly, our studies demonstrate that it is crucial to incorporate both positive and negative reciprocity motives when studying the effects of LMX. Implications of these results for social exchange theory, LMX, and the broader literature of (self- and other-serving) unethical behavior are discussed.

## Introduction

Unethical behavior, despite being costly and detrimental for organizations (Giacalone and Promislo, 2010), is quite prevalent and is expected to become even more prevalent in the future (Rickman and Witt, 2007). Research examining the predictors and mechanisms of unethical behavior has greatly increased in the last decades (Treviño et al., 2014). Several studies have investigated what leaders, as central gatekeepers of appropriate conduct, can do to prevent unethical behavior among their followers (Treviño and Brown, 2005; Brown and Treviño, 2006; Kalshoven et al., 2016). The quality of the leader–member exchange (LMX) relationship between leaders and followers (Dienesch and Liden, 1986; Graen and Uhl-Bien, 1995; Settoon et al., 1996) appears to play a prominent role in preventing employee unethical behavior. Under the assumption that followers aim to positively reciprocate high-quality LMX relationships, initial studies have demonstrated that LMX is *negatively* related to self-serving behaviors such as deviance, counterproductive work behaviors, and withdrawal (Gerstner and Day, 1997; El Akremi et al., 2010; Liu et al., 2013; Jawahar et al., 2018).

Interestingly, however, under the same positive reciprocity assumption, more recent studies have demonstrated that LMX is *positively* related to other-serving undesirable behaviors as pro-leader unethical behavior (Bryant and Merritt, 2019). In these recent studies, followers are assumed to use pro-leader unethical behaviors to positively reciprocate the benefits they receive from their leaders in high-quality LMX relationships. As such, this positive reciprocity mechanism is used in research to explain both why high-quality LMX relationships *demotivate* (El Akremi et al., 2010; Liu et al., 2013; Jawahar et al., 2018) and *motivate* (Umphress et al., 2010; Bryant and Merritt, 2019) unethical behaviors. That is, the negative effects of LMX on self-serving unethical behavior and its positive effects on other-serving unethical behavior are assumed to be driven by the same motivational mechanism of positive reciprocity. We argue that this one-sided focus on positive reciprocity motives derived from high-quality LMX relationships foregoes the role of negative reciprocity motives sparked by low-quality LMX relationships (Uhl-Bien and Maslyn, 2003). While the (lack of) benefits to an exchange may be an important motivator for followers, it seems equally feasible that negative reciprocity either *motivates* self-serving unethical behavior or *demotivates* other-serving unethical behavior. Exploring these underlying differences between positive and negative reciprocity motives is imperative in understanding why, when, and how LMX relates to unethical behaviors that serve either the follower or others, and may be crucial in developing interventions that aim to reduce all types of unethical behaviors.

Drawing from the social exchange theory (Gouldner, 1960; Blau, 1964) and the LMX theory (Liden and Graen, 1980; Graen and Uhl-Bien, 1995), we argue that the quality of the LMX relationship motivates followers to either positively or negatively reciprocate this relationship, leading them to consider either pro-leader or pro-self unethical behavior. We provide empirical evidence for our proposed conceptual model across an experimental study and a time-split field study. By presenting a coherent framework that explains when (i.e., high- vs. low-quality LMX), why (i.e., positive vs. negative reciprocity), and how (i.e., pro-leader vs. pro-self unethical behavior) individuals consider unethical behavior as a means of reciprocating exchanges with their leader, we contribute to the literatures on social exchange theory (e.g., Gouldner, 1960; Blau, 1964), LMX (e.g., Dienesch and Liden, 1986; Graen and Uhl-Bien, 1995; Settoon et al., 1996), and (self- and other-serving) unethical behavior (e.g., Umphress et al., 2010; Treviño et al., 2014; Bryant and Merritt, 2019). Our specific contributions are fourfold. First, although previous research has demonstrated that LMX can both *demotivate* (El Akremi et al., 2010; Liu et al., 2013; Jawahar et al., 2018) and *motivate* (Umphress et al., 2010; Bryant and Merritt, 2019) unethical behaviors, our study is among the first to directly contrast these motivations in a single study. Second, although previous research has certainly suggested that reciprocal social exchange mechanisms play a crucial role in motivating unethical behavior (e.g., Umphress et al., 2010; Umphress and Bingham, 2011; Miao et al., 2013; Effelsberg et al., 2014; Effelsberg and Solga, 2015; Kalshoven et al., 2016; Kong, 2016; Bryant and Merritt, 2019; Lee et al., 2019), none of these have explicitly tested how reciprocity motives facilitate the relationship between LMX and unethical behavior. This is especially important in light of recent criticisms of LMX research that the social exchange component is often ignored altogether (Gottfredson et al., 2020). Third, although LMX can motivate both positive and negative reciprocity motives, research tends to limit itself to the positive aspect (Uhl-Bien and Maslyn, 2003). Our study demonstrates that positive and negative reciprocity entail different motives that predict different intended unethical behaviors in meaningfully different ways. Finally, given the prevalence and importance of LMX relationships (Henderson et al., 2009) and the destructive nature of unethical behavior (Giacalone and Promislo, 2010), our study offers practitioners an important consideration when developing LMX relationships.

## Leader–Member Exchange Theory

The LMX theory (Dienesch and Liden, 1986; Graen and Uhl-Bien, 1995; Settoon et al., 1996) explains how the relationships between leaders and followers develop (role theory; Graen, 1976) and how this relationship formation determines interactions between leaders and followers (social exchange theory; Gouldner, 1960; Blau, 1964). According to the LMX theory, leaders and followers go through role-taking and role-making processes to determine what both parties can expect from one another (for a review, see Dulebohn et al., 2012). Leaders and followers either develop low-quality economic exchange relationships where leaders reward followers for performing the duties as specified in their work contract, or develop more high-quality social exchange relationships where leaders additionally exchange affect, loyalty, contribution, and professional respect (Liden and Maslyn, 1998). The quality of the LMX relationship refers to the extent to which followers perceive that an exchange of social resources is absent (i.e., a low-quality LMX relationship) or present (i.e., a high-quality LMX relationship) (Lord and Maher, 1991; Engle and Lord, 1997).

The basic use of social exchange theory in LMX is as follows. As leaders have limited resources to establish exchange relationships with followers (Liden and Graen, 1980; Graen and Uhl-Bien, 1995), they tend to differentiate among followers and develop high-quality social exchange relationships with some followers, while maintaining low-quality economic exchange relationships with others. This differentiation implies that followers consider both the quality of their own LMX relationship and that of their peers to determine the extent to which they are obligated to engage in positive reciprocity, where benefit is returned with benefit, or in negative reciprocity, where harm is returned with harm (Gouldner, 1960; Blau, 1964). Followers who perceive a higher-quality LMX relationship with their leader will feel privileged relative to peers with lower-quality LMX relationships and will interpret their relatively high-quality social exchanges with their leader as a unique benefit (Henderson et al., 2009). This unique benefit then instills a positive reciprocity motive among these followers (Gouldner, 1960; Perugini et al., 2003; Brandes and Franck, 2012), where followers are motivated to return the benefit they received with equal benefit. In contrast, followers who perceive a lower-quality LMX relationship will feel deprived relative to peers with higher-quality LMX relationships, which will lead to perceptions of unfair treatment (Folger and Martin, 1986; Masterson et al., 2000) and dissatisfaction with their leader (McClane, 1991). This deprivation then instills a negative reciprocity motive among these followers (Uhl-Bien and Maslyn, 2003; Eisenberger et al., 2004), which may motivate them to return the harm they received with equal harm.

Although the duality of positive and negative reciprocity is a necessary element of LMX relationships, most studies on LMX are limited to positive reciprocity (Uhl-Bien and Maslyn, 2003). Specifically, LMX research typically uses the positive reciprocity route to argue for relationships with undesirable behavior, both of the kind that benefits employees themselves (El Akremi et al., 2010; Liu et al., 2013; Jawahar et al., 2018) and those that benefit others (Umphress et al., 2010; Bryant and Merritt, 2019). Although certain studies have demonstrated that general positive reciprocity beliefs moderate the effects of LMX and organizational identification on unethical behavior (Umphress et al., 2010; Bryant and Merritt, 2019), no studies known to us have empirically investigated the intervening effects of positive reciprocity motivated by high-quality LMX relationships, let alone intervening effects of negative reciprocity motivated by low-quality LMX relationships. This is problematic, as not differentiating between positive and negative reciprocity confounds the potentially different motives that employees may have to engage in different unethical behaviors, especially different unethical behaviors that serve to benefit different parties. In what follows, we will contrast pro-leader with pro-self unethical behaviors and argue how engaging in these distinct behaviors causes employees to differentially satisfy either positive or negative reciprocity motives.

### Unethical Behavior as Means of Positive and Negative Reciprocity

Unethical behavior is typically defined as any act that is “either illegal or morally unacceptable to the larger community” (Jones, 1991, p. 367). The majority of studies has focused on unethical behavior that serves to benefit oneself (Umphress et al., 2010; Umphress and Bingham, 2011). Recent research, however, has demonstrated that individuals also engage in unethical behavior to benefit others, including their organizations (Umphress et al., 2010; Umphress and Bingham, 2011), groups (Thau et al., 2015), and even leaders (Johnson and Umphress, 2018; Mesdaghinia et al., 2018; Bryant and Merritt, 2019). Benefits, in these contexts, are typically represented by the extent to which unethical behavior allows one to more effectively or efficiently ensure desirable outcomes (Schweitzer et al., 2004; Ordóñez et al., 2009; Welsh and Ordóñez, 2014). Within this study, we distinguish *pro-leader* and *pro-self* unethical behavior by defining them as follower “actions that are intended to promote the effective functioning of (the follower’s leader *or* the follower him-/herself) and violate core societal values, mores, laws, or standards of proper conduct” (cf. Umphress and Bingham, 2011, p. 622). As is the case with similar constructs, it is important to stress that benefits are *intended*, regardless of whether the beneficiary actually benefits from the unethical behavior (Sackett, 2002; Umphress et al., 2010).

Engaging in unethical behavior is typically associated with various negative consequences and costs, including direct or indirect punishment and damage to their reputation or moral identity (Becker, 1968; Gino and Margolis, 2011; Mulder et al., 2015). Accordingly, followers tend to be inhibited from engaging in unethical behavior (Jordan and Monin, 2008) and need to be released of these inhibitions before they can engage in them (Treviño et al., 2014), both to benefit oneself (e.g., Vriend et al., 2016) and others (e.g., Chen et al., 2016). Ethical inhibitions can be released when the perceived benefits of unethical behavior outweigh its perceived costs (Becker, 1968; Lewicki, 1983). For pro-self unethical behavior, this is typically the case when personal gains can be ensured (e.g., Brief et al., 2001; Gino and Margolis, 2011) or when relationships can be maintained (El Akremi et al., 2010; Liu et al., 2013; Jawahar et al., 2018). Within the broader tradition of unethical pro-organizational behavior research, pro-leader unethical behavior benefits may include the opportunity to satisfy needs for affiliation (Thau et al., 2015), strengthen relational ties (Miao et al., 2013; Effelsberg et al., 2014; Johnson and Umphress, 2018), or enact reciprocity beliefs (Umphress et al., 2010; Bryant and Merritt, 2019; Wang et al., 2019).

#### Positive Reciprocity Motives and Pro-leader Unethical Behavior

We argue that pro-leader unethical behavior will be able to satisfy the positive reciprocity motive instilled by high-quality LMX relationships. High-quality LMX relationships create an obligation for followers to positively reciprocate the benefit they receive from their leaders by engaging in actions that benefit their leaders in return (Gouldner, 1960; Uhl-Bien and Maslyn, 2003; Brandes and Franck, 2012). Followers that fulfill this reciprocity motive by engaging in pro-leader unethical behavior gain no direct benefit themselves. Instead, for the follower, the prime functionality of the pro-leader unethical behavior is that the benefit is directly bestowed upon their leader (Mesdaghinia et al., 2018). Despite the lack of direct self-benefits accrued through pro-leader unethical behavior, however, followers do risk its consequences (cf. Becker, 1968; Gino and Margolis, 2011; Mulder et al., 2015). This indicates that followers would be willing to go through great lengths, at potentially great costs, to benefit their leader.

Although followers may be inhibited to engage in pro-leader unethical behavior (cf. Jordan and Monin, 2008; Chen et al., 2016), previous research has established that strong relational ties may release such ethical inhibitions (Umphress et al., 2010; Umphress and Bingham, 2011; Miao et al., 2013; Thau et al., 2015; Johnson and Umphress, 2018). High-quality LMX relationships are characterized by mutual affect, loyalty, and liking (Liden and Maslyn, 1998), which signal strong relational ties. Such strong relational ties can encourage followers to resort to pro-leader unethical behavior. They may, for example, exaggerate successes and lie about wrongdoings of their leader to others, intending to benefit or protect the leader and maintain the high-quality LMX relationship. Thus, as high-quality LMX relationships obligate followers to positively reciprocate the benefits they receive from their leader (Gouldner, 1960; Brandes and Franck, 2012), pro-leader unethical behavior allows followers to satisfy this obligation (cf. Umphress and Bingham, 2011).

#### Negative Reciprocity Motive and Pro-self Unethical Behavior

We argue that pro-self unethical behavior satisfies the negative reciprocity motive instilled by low-quality LMX relationships. Low-quality LMX relationships are characterized as economic exchange relationships, in which followers are expected to adhere to the formal obligations, rules, standards, and norms as stipulated in their work contract (Liden and Maslyn, 1998). Similarly, leaders are expected to hold their followers accountable for violations of these contract obligations (Treviño and Brown, 2005; Brown and Mitchell, 2010). Followers with a low-quality LMX relationship are likely to feel deprived relative to peers who have higher-quality LMX relationships (Henderson et al., 2009), instilling a negative reciprocity motive in them (Uhl-Bien and Maslyn, 2003; Eisenberger et al., 2004). When followers engage in pro-self unethical behavior, they directly violate the formal obligations, rules, standards, and norms that their leaders are holding them accountable for. This signals that followers’ gains obtained by their unethical behavior are more important to them than adhering to the rules stipulated by their leader. Pro-self unethical behavior therefore satisfies a negative reciprocity motive by directly degrading and corrupting the economic exchanges that they are expected to maintain as stipulated by the formal work contract. Pro-self unethical behavior can therefore be perceived as an effective means through which followers can reciprocate the felt unfair treatment and restore the balance in the relationship with their leader. Typical examples of pro-self unethical behaviors driven by a negative reciprocity motive in LMX relationships include both leader- and organization-directed deviance (El Akremi et al., 2010; Liu et al., 2013), counterproductive work behavior (Jawahar et al., 2018), withdrawal behaviors (Gerstner and Day, 1997), and illegitimate acts such as exaggerating one’s successes or illegally appropriating resources.

## Study 1

The purpose of Study 1 was to investigate whether LMX indeed differentially relates to pro-leader and pro-self unethical behaviors and whether these effects are indeed driven by positive and negative reciprocity motives. More specifically, we conducted an experimental study to assess, first, whether low-quality LMX relationships motivate more pro-self than pro-leader unethical intentions and whether high-quality LMX relationships motivate more pro-leader than pro-self unethical intentions. Second, we examined whether positive reciprocity motives explain why high-quality LMX relationships are more likely to motivate pro-leader than pro-self unethical intentions and negative reciprocity motives explain why low-quality LMX relationships are more likely to motivate pro-self than pro-leader unethical intentions.

### Materials and Methods

#### Participants

One hundred and sixty-four United States residents (*M*_age_ = 31.75, *SD*_age_ = 11.10, 40.85% female) were recruited through Mturk. We told participants that we were investigating how personality influences decision-making. Participants were provided with $0.50 for their participation.

#### Procedure

We randomly assigned participants to one of four experimental conditions in a 2 (LMX: high vs. low) × 2 (type of unethical behavior: pro-leader vs. pro-self) between-subjects design. We first provided participants with either a high-LMX or a low-LMX scenario (adapted from Bhal and Dadhich, 2011) (low LMX between brackets in *italics*):

“*You and your supervisor (do not) get along very well. You (do not) like your supervisor as a person very much, and you (do not) like working with your supervisor. The two of you just (do not) get along. You have the feeling that your supervisor does not only treat (only treats) you as an employee, but also (and not) as a unique person, and that you can (not) go to your supervisor with personal wishes and problems. Your relationship is based on mutual trust (your formal work contract). Because your supervisor is (not) willing to do something extra for you, you are also (not) willing to do more than strictly necessary.*”

##### Positive and negative reciprocity motives

After the LMX manipulation, we used a shortened (cf. Caliendo et al., 2012; Egloff et al., 2013) version of Perugini et al.’s (2003) measurement instrument to assess the extent (1 = *fully disagree*, 7 = *fully agree*) to which participants would have positive and negative reciprocity motives in relationship to the supervisor depicted in the scenario. Positive reciprocity motive (α = 0.86) was assessed by the items: “If my supervisor does me a favor, I am prepared to return it,” “I go out of my way to help my supervisor who has been kind to me before,” and “I am ready to undergo personal costs to help my supervisor who helped me before.” Negative reciprocity motive (α = 0.85) was assessed by the items: “If my supervisor causes me to suffer a serious wrong, I will take revenge as soon as possible, no matter what the cost,” “If my supervisor puts me in a difficult position, I will do the same to my supervisor,” and “If my supervisor offends me, I will offend my supervisor back.”

##### Pro-leader and pro-self unethical intentions

After inquiring about their reciprocity motives, depending on their assigned condition, we asked participants to indicate either the extent (1 = *fully disagree*, 7 = *fully agree*) to which they would engage in pro-leader or pro-self unethical behavior. Pro-leader unethical intention (α = 0.91) was assessed by the items: “If it would help my supervisor, I would misrepresent the truth to make my supervisor look good,” “If it would help my supervisor, I would exaggerate the truth about my supervisor’s successes to others,” and “If it would benefit my supervisor, I would withhold negative information about my supervisor to others.” Pro-self unethical intention (α = 0.78) was assessed by the items: “If it would help me, I would misrepresent the truth to make me look good,” “If it would help me, I would exaggerate the truth about my successes to others,” and “If it would benefit me, I would withhold negative information about myself to others.” These items were based on similar items developed by Umphress et al. (2010) and Johnson and Umphress (2018)^1^.

We piloted these items for discriminant validity in a sample of 221 employed United States residents (*M*_age_ = 31.29, *SD*_age_ = 9.85, 33.94% female) recruited through Mturk. An exploratory factor analysis revealed two distinct pro-leader and pro-self unethical intention factors that together explained 78.99% of the variance. Pro-leader and pro-self unethical intentions were positively correlated, *r*(220) = 0.42, *p* < 0.001, which is comparable to correlations between similar constructs (e.g., the meta-analytic correlation between interpersonal and organizational deviance, ρ = 0.62, as reported by Berry et al., 2007). These results confirm that intended pro-leader and pro-self unethical behaviors are distinct and measurable constructs.

##### Manipulation check

To check our LMX manipulation, we used Liden and Maslyn’s (1998) 11-item measurement instrument to assess how participants perceived the quality of the relationship with the supervisor as described in the scenario (1 = *fully disagree*, 7 = *fully agree*; α = 0.99). Example items include: “I like my supervisor very much as a person,” “My supervisor would come to my defense if I were ‘attacked’ by others,” and “I am willing to apply extra efforts, beyond those formally required to further the interests of my supervisor.”

##### Control variables

We controlled for gender and age. Substantive conclusions drawn from the results are similar both with and without control variables.

### Results

Descriptive statistics and intercorrelations are reported in Table 1. The significant correlation between the LMX manipulation and the LMX manipulation check, *r*(164) = 0.87, *p* < 0.001, indicates that LMX was successfully manipulated.

**TABLE 1:** Descriptive statistics and intercorrelations (Study 1).

Variable	*M*	*SD*	1	2	3	4	5	6	7	8
1. Gender (0 = male, 1 = female)	1.41	0.49	–							
2. Age (years)	31.75	11.10	0.18*	–						
3. Leader–member exchange manipulation (0 = low, 1 = high)	0.51	0.50	–0.10	–0.01	–					
4. Unethical behavior manipulation (0 = pro-leader, 1 = pro-self)	0.49	0.50	0.05	–0.12	–0.05	–				
5. Leader–member exchange manipulation check	4.06	2.17	–0.08	0.05	0.87***	0.00	(0.99)			
6. Positive reciprocity motive	4.56	1.76	–0.03	0.03	0.63***	0.08	0.74***	(0.86)		
7. Negative reciprocity motive	3.32	1.61	−0.17*	−0.27***	−0.35***	0.03	−0.42***	−0.36***	(0.85)	
8. Unethical intention	3.29	1.64	–0.14	–0.11	–0.06	0.26***	–0.06	–0.07	0.28***	(0.85)

N = 164. Cronbach’s alphas between parentheses on the diagonal. * p < 0.05, *** p < 0.001.

#### The Effect of LMX on Unethical Intentions

**FIGURE 1:**
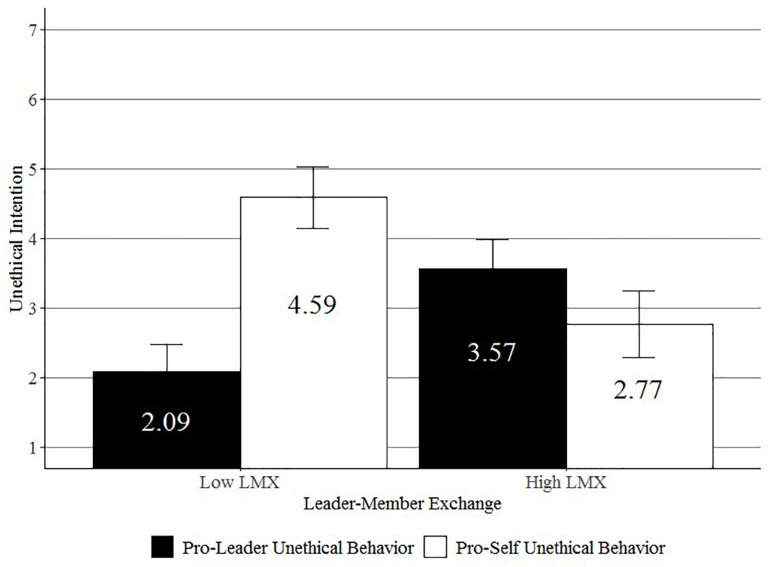
Effect of LMX on unethical intentions (Study 1).

A 2 (LMX: low vs. high) × 2 (type of unethical behavior: pro-leader vs. pro-self) ANOVA on unethical intention revealed no significant main effect of LMX, *F*(1, 160) = 0.79, *ns*, a significant main effect of type of unethical behavior, *F*(1, 160) = 15.25, *p* < 0.001, and a significant interaction effect, *F*(1, 160) = 59.83, *p* < 0.001. Additional contrast analyses revealed that participants in the high LMX condition had higher pro-leader (*M* = 3.57, *SD* = 1.34) than pro-self (*M* = 2.77, *SD* = 1.48) unethical intentions, *t*(160) = 2.66), *p* < 0.01, and that participants in the low LMX condition had higher pro-self (*M* = 4.59, *SD* = 1.42) than pro-leader (*M* = 2.09, *SD* = 1.20) unethical intentions, *t*(160) = 8.25, *p* < 0.001. These results are illustrated in Figure 1.

#### The Mediating Role of Reciprocity Motives

To assess the extent to which reciprocity motives mediate the indirect relationship between LMX and unethical intention, we conducted moderated-mediation regression path analyses using lavaan 0.6-5 in *R* (Rosseel, 2012). Specifically, we followed Model 14 moderated-mediation procedures as described by Hayes (2013), which allowed us to assess our theoretical rationale that the effects of LMX on intended unethical behavior are driven by positive and negative reciprocity motives, and that these motives dictate what type of unethical intention is elicited. Standardized results of the regression path analysis and conditional indirect effects are reported in Table 2.

**TABLE 2:** Standardized coefficients for regression path analysis and conditional indirect effects (Study 1).

Variable	Positive reciprocity motive	Negative reciprocity motive	Unethical intention
**Regression path analysis**			
Gender	0.03 (0.06)	−0.16*(0.07)	−0.12(0.07)
Age	0.04 (0.06)	−0.24***(0.07)	−0.06(0.07)
Leader–member exchange (manipulation)	0.63***(0.06)	−0.37***(0.07)	0.09 (0.08)
Type of unethical behavior (manipulation)			0.26***(0.06)
Positive reciprocity motives			−0.03(0.08)
Negative reciprocity motives			0.24***(0.07)
Type of unethical behavior (manipulation) × Positive reciprocity motives			−0.34***(0.07)
Type of unethical behavior (manipulation) × Negative reciprocity motives			0.24***(0.07)
*R*^2^	0.40	0.22	0.37
**Conditional indirect effects**			
Leader–member exchange (manipulation) –> Positive reciprocity motive > Unethical intention (pro-leader)			0.19**(0.07)
Leader–member exchange (manipulation) –> Positive reciprocity motive > Unethical intention (pro-self)			−0.24***(0.07)
Leader–member exchange (manipulation) –> Negative reciprocity motive > Unethical intention (pro-leader)			−0.00(0.04)
Leader–member exchange (manipulation) –> Negative reciprocity motive > Unethical intention (pro-self)			−0.18***(0.05)

N = 164. Standard errors between parentheses. * p < 0.05, ** p < 0.01, *** p < 0.001.

First, results indicate that our LMX manipulation has a positive effect on positive reciprocity motive (β = 0.63, *p* < 0.001) and a negative effect on negative reciprocity motive (β = 0.37, *p* < 0.01), which supports our premise that the quality of LMX relationship dictates reciprocity motives. Second, results indicate that non-significant effect of positive reciprocity motive on unethical intention (β = -0.03, *ns*) is moderated by the type of unethical behavior (β = -0.34, *p* < 0.001), such that positive reciprocity motive is positively related to pro-leader unethical behavior (β = 0.30, *p* < 0.001) and negatively related to pro-self unethical behavior (β = -0.37, *p* < 0.001). Third, results indicate that negative reciprocity motive has a significant effect on unethical intention (β = 0.24, *p* < 0.001) that is conditional upon the type of unethical behavior (β = 0.24, *p* < 0.001), such that negative reciprocity motive is unrelated to pro-leader unethical behavior (β = 0.00, *ns*) and positively related to pro-self unethical behavior (β = 0.48, *p* < 0.001). Finally, conditional indirect effects indicate that high-quality LMX relationships, through positive reciprocity motives, increase pro-leader unethical intention (β = 0.19, *p* < 0.001) and decrease pro-self unethical intention (β = −0.24, *p* < 0.001), and that low-quality LMX relationships, through negative reciprocity motives, do not affect pro-leader unethical behavior (β = −0.00, *ns*), but do increase pro-self unethical intention (β = −0.18, *p* < 0.001).

### Discussion

The results of Study 1 provide us with a first indication that high-quality LMX relationships can motivate pro-leader unethical behavior to satisfy positive reciprocity motives and that low-quality LMX relationships can motivate pro-self unethical behavior to satisfy negative reciprocity motives. Despite its merits, however, Study 1 has several limitations that prevent us from drawing too strong conclusions with respect to our expected relationships. First, although previous research has also manipulated LMX by means of scenarios (Bhal and Dadhich, 2011), actual LMX relationships develop over a longer period of time (Liden and Graen, 1980; Dienesch and Liden, 1986; Graen and Uhl-Bien, 1995), which is something that is difficult to capture through experimental manipulations. Second, although our diverse sample allows us to generalize across a multitude of occupations, organizations, and industries, it does not allow us to generalize across nationalities. Research has demonstrated that the effects of LMX may be culturally dependent, especially when aspects of ethics and fairness are concerned (Rockstuhl et al., 2012). Similarly, national culture also has a substantial influence on ethical decision-making (Westerman et al., 2007). Accordingly, taken together, it would be useful to assess our expected relationships in a different (national) context where the quality of the LMX relationship has been able to mature over time.

## Study 2

The purpose of Study 2 was to replicate the mediating mechanisms of Study 1 in a different cultural setting using a time-split field study among followers that have actually been able to develop an LMX relationship with their leaders over time.

### Materials and Methods

#### Sample and Procedure

We collected three-wave time-split data from followers from different companies from various industries in the Netherlands, including construction, education, healthcare, local government, and retail, among others. We invited 480 followers to participate in our study for Wave 1. A total of 366 followers (76.25%) completed the first questionnaire. Two months after inviting them to participate in the first wave, we invited these 366 followers to participate in Wave 2. A total of 330 followers (90.16%) completed the second questionnaire. Four months after inviting them to participate in the second wave, we invited these followers to participate in Wave 3. A total of 269 followers (81.52%) completed the third questionnaire. Of these 269 followers, 120 were male and 149 female, with an average age of 43.77 years (*SD* = 11.74, *range* = 17–65) and organizational tenure of 11.47 years (*SD* = 9.59, *range* = 0–39). Most followers held a lower (99) or higher (110) vocational degree.

#### Measures

We measured all scales on a five-point Likert scale, ranging from *strongly disagree* (1) to *strongly agree* (5). As the questionnaires were in Dutch, we translated the scales from English to Dutch using a back-translation procedure (Brislin, 1970).

##### LMX

We assessed LMX (α = 0.87) in the first wave by means of the 11 items developed by Liden and Maslyn (1998) described earlier.

##### Positive and negative reciprocity motives

We assessed positive (α = 0.79) and negative (α = 0.88) reciprocity motives in the second wave by means of the items developed by Perugini et al. (2003) described earlier.

##### Pro-leader and pro-self unethical intentions

We assessed intended pro-leader (α = 0.76) and pro-self (α = 0.81) unethical behavior in the third wave by means of the items used previously.

##### Control variables

We controlled for gender, age, tenure with organization, tenure with leader, and number of hours weekly worked under contract. Substantive conclusions drawn from the results are similar both with and without control variables.

#### Convergent and Divergent Validity and Common Method Bias Considerations

We used lavaan 0.6-5 in *R* (Rosseel, 2012) to assess the convergent and discriminant validities for the suggested measurement model, to compare this with various alternative measurement models, and to assess the extent of the common method bias. We first estimated a baseline measurement model in which all items loaded freely on their focal and designated construct without any cross-loadings. This baseline measurement model provided an unacceptable fit to the data [χ^2^(220) = 770.66, *RMSEA* = 0.10 [0.09–0.10], *CFI* = 0.82, *TLI* = 0.79] but was superior to models in which we collapsed LMX and positive reciprocity, Δχ(4) = 247.20, *p* < 0.001, LMX and negative reciprocity Δχ(4) = 444.39, *p* < 0.001, positive and negative reciprocity, Δχ(4) = 449.08, *p* < 0.001, and pro-leader and pro-self unethical intentions, Δχ(4) = 144.26, *p* < 0.001. We then estimated a model in which we included an uncorrelated methods factor (cf. Podsakoff et al., 1990). Adding this uncorrelated methods factor significantly improved the model fit over our baseline model, Δχ(20) = 241.86, *p* < 0.001, indicating that common method variance is present. Squaring the standardized factor loadings of the items with the uncorrelated common method factor indicated that 5.24% of the variance can be attributed to a common method.

We then followed the procedures outlined by Williams and McGonagle (2016) to assess the degree of common method variance present in our study and the extent of its effects on (interrelations between) substantive variables. First, we compared the common methods factor model with a common methods factor model in which the substantive factor intercorrelations were constrained to be equal to those of the baseline model. The fit between the restricted and unrestricted models was not significantly different, Δχ(10) = 0.46, *ns*, indicating that the presence of common method variance does not influence the interrelationships between the substantive factors. Second, we calculated the substantive and method reliability for all five substantive factors. Results demonstrate that LMX, positive and negative reciprocity, and pro-leader and pro-self unethical intentions have acceptable substantive reliabilities (0.82, 0.79, 0.89, 0.80, 0.81) and relatively low method reliabilities (0.07, 0.00, 0.00, 0.01, 0.00), indicating that the presence of common method variance does not influence the substantive meaning of the substantive factors.

Finally, we explored potential sources of the unacceptable fit of the baseline model. Supplementary analyses revealed that removing the 11 LMX items from the baseline model resulted in a good fit to the data [χ^2^(48) = 158.16, *RMSEA* = 0.09 [0.08–0.11], *CFI* = 0.93, *TLI* = 0.91], substantially improving the model fit relative to the baseline model (Δ*CFI* = 0.11, Δ*TLI* = 0.12). This indicates that our measurement instrument for LMX, which is multidimensional in nature (Liden and Maslyn, 1998), may be the primary culprit for the poor fit of our baseline model. To verify this, we employed the internal-consistency approach to parceling (Kishton and Widaman, 1994; Little et al., 2002). A model in which we parceled the 11 LMX items into four parcels based on their underlying dimensions, affect, contribution, loyalty, and professional respect also resulted in a good fit to the data [χ^2^(94) = 238.48, *RMSEA* = 0.08 [0.06–0.09], *CFI* = 0.92, *TLI* = 0.90], substantially improving the model fit relative to the baseline model (Δ*CFI* = 0.11, Δ*TLI* = 0.12). Although these results may imply that the measurement instrument for LMX may suffer from poor reliability in our sample, this should have limited consequences for our statistical analyses (cf. Little et al., 2002).

### Results

Descriptive statistics and intercorrelations are reported in Table 3.

**TABLE 3:** Descriptive statistics and first-order intercorrelations (Study 2).

Variable	*M*	*SD*	1	2	3	4	5	6	7	8	9	10
1. Gender (0 = male, 1 = female)	0.55	0.50	–									
2. Age (years)	43.77	11.74	–0.04	–								
3. Tenure with organization (years)	11.47	9.59	–0.08	0.59***	–							
4. Tenure with leader (years)	4.35	5.30	−0.19**	0.20**	0.33***	–						
5. Number of contractual hours (per week)	30.84	8.92	−0.56***	0.11	0.07	0.12	–					
6. Leader–member exchange	3.90	0.55	0.05	–0.09	–0.07	0.01	0.03	(0.87)				
7. Positive reciprocity motive	3.14	0.86	0.00	−0.23***	−0.16**	–0.01	–0.07	0.18**	(0.79)			
8. Negative reciprocity motive	1.59	0.71	−0.23***	0.03	0.14*	0.09	0.21***	−0.16**	0.17**	(0.88)		
9. Pro-leader unethical intention	2.78	0.89	−0.14*	–0.11	–0.11	0.01	0.14*	0.12	0.26***	0.11	(0.76)	
10. Pro-self unethical intention	2.45	0.87	−0.19**	–0.08	–0.10	–0.01	0.17**	–0.02	0.16**	0.24***	0.61***	(0.81)

N = 269. Cronbach’s alphas between parentheses on the diagonal. * p < 0.05, ** p < 0.01, *** p < 0.001.

To assess the mediating role of positive and negative reciprocity motives, we conducted mediation regression path analyses using lavaan 0.6-5 in *R* (Rosseel, 2012). Specifically, we (1) regressed positive and negative reciprocity motives on LMX and the control variables and (2) regressed pro-leader and pro-self unethical intentions on positive and negative reciprocity motives, LMX, and the control variables. Standardized results of the regression path analysis and conditional indirect effects are reported in Table 4.

**TABLE 4:** Standardized coefficients for regression path analysis and indirect effects (Study 2).

Variable	Positive reciprocity motive	Negative reciprocity motive	Pro-leader unethical intention	Pro-self unethical intention
**Regression path analysis**				
Gender	−0.06(0.07)	−0.14(0.07)	−0.08(0.07)	−0.11(0.07)
Age (years)	−0.18*(0.07)	−0.10(0.07)	−0.02(0.07)	−0.01(0.07)
Tenure with organization (years)	−0.06(0.08)	0.15*(0.07)	−0.08(0.07)	−0.11(0.07)
Tenure with leader (years)	0.04 (0.06)	0.02 (0.06)	0.01 (0.06)	−0.02(0.06)
Number of contractual hours (per week)	−0.09(0.07)	0.13 (0.07)	0.10 (0.07)	0.08 (0.07)
Leader–member exchange	0.16**(0.06)	−0.16**(0.06)	0.08 (0.06)	−0.01(0.06)
Positive reciprocity motive			0.23***(0.06)	0.12*(0.06)
Negative reciprocity motive			0.05 (0.06)	0.20**(0.06)
*R*^2^	0.09	0.11	0.11	0.10
**Indirect effects**				
Leader–member exchange –> Positive reciprocity motive > Pro-leader unethical intention				0.04*(0.02)
Leader–member exchange –> Positive reciprocity motive > Pro-Self unethical intention				0.02 (0.01)
Leader–member exchange –> Negative reciprocity motive > Pro-leader unethical intention				−0.01(0.01
Leader–member exchange –> Negative reciprocity motive > Pro-self unethical intention				−0.03*(0.01)

N = 269. Standard errors between parentheses. * p < 0.05, ** p < 0.01, *** p < 0.001.

First, the results indicate that LMX has a positive effect on positive reciprocity motive (β = 0.16, *p* < 0.01) and a negative effect on negative reciprocity motive (β = -0.16, *p* < 0.01). Second, the results indicate that positive reciprocity motive has a positive effect on both pro-leader (β = 0.23, *p* < 0.001) and pro-self (β = 0.12, *p* < 0.05) unethical intentions. Third, the results indicate that negative reciprocity motive has no significant effect on pro-leader unethical intention (β = 0.05, *ns*), but does have a significant positive effect on pro-self unethical intention (β = 0.20, *p* < 0.01). Finally, conditional indirect effects indicate that the relationship between LMX and pro-leader unethical intention is primarily driven by positive reciprocity motives (β = 0.04, *p* < 0.05) and not negative reciprocity motives (β = -0.01, *ns*), and that the relationship between LMX and pro-self unethical intention is primarily driven by negative reciprocity motives (β = -0.03, *p* < 0.05) and not positive reciprocity motives (β = 0.02, *ns*).

### Discussion

The results of Study 2 provide further support for our premise that both high- and low-quality LMX relationships can motivate followers to engage in unethical behavior, albeit for different reasons. More specifically, followers with a high-quality LMX relationship are motivated to engage in pro-leader unethical behavior to satisfy negative reciprocity motives, and followers with a low-quality LMX relationship are motivated to engage in pro-self unethical behavior to satisfy negative reciprocity motives. In contrast to Study 1, however, we did not find a significant indirect relationship from LMX to pro-self unethical intention that is mediated by positive reciprocity motive.

## General Discussion

In this investigation, we took a social exchange perspective to identify when, why, and how leaders may unintendedly motivate followers to consider unethical behavior that either serves the leader or the self. Across two studies, we find compelling empirical evidence to support our expectation that high-quality LMX relationships motivate pro-leader unethical intention to satisfy positive reciprocity motives and that low-quality LMX relationships motivate pro-self unethical intention to satisfy negative reciprocity motives. The diverse nature of the studies allows us to generalize these findings across a wide variety of occupations, organizations, industries, and even cultures. Furthermore, our experimental setup in Study 1 and the time-split nature of Study 2 provide further credence to the causal direction of our expected effects.

In addition to the expected effects, there was an unexpected and inconsistent cross-effect of reciprocity on intended unethical behavior across the studies. Specifically, while we found in Study 1 that positive reciprocity motive is *negatively* related to intended pro-self unethical behavior, in Study 2, we found that this relationship is *positive*. These inconsistent cross-effects of positive reciprocity motive may be evidence for a cultural dependency effect of LMX (Rockstuhl et al., 2012) and ethical decision-making (Westerman et al., 2007). On a more general level, however, this could indicate that there is more to the relationship between reciprocity motive and unethical behavior than we envisioned. The unexpected positive cross-effect of positive reciprocity on intended pro-self unethical behavior in Study 1, for example, could indicate that a positive reciprocity motive (i.e., doing good) may license followers to engage in intended unethical behavior for their own benefit (i.e., doing bad) (cf. Sachdeva et al., 2009). The unexpected negative cross-effect of positive reciprocity on intended pro-self unethical behavior in Study 2, on the other hand, could, for example, indicate that followers may not only be concerned with reciprocating established exchange relationships (i.e., paying back), but also be concerned with developing future exchange relationships over time (i.e., paying forward) (Korsgaard et al., 2010).

### Theoretical Implications

Our theoretical and empirical findings have implications for various streams of literature, particularly on social exchange theory (e.g., Gouldner, 1960; Blau, 1964), LMX (e.g., Dienesch and Liden, 1986; Graen and Uhl-Bien, 1995; Settoon et al., 1996), and (self- and other-serving) unethical behavior (e.g., Umphress et al., 2010; Treviño et al., 2014; Bryant and Merritt, 2019). First, our findings have implications for the notion that leaders, as gatekeepers of appropriate conduct, are tasked with preventing self-interested unethical behavior among their followers (Treviño and Brown, 2005; Brown and Treviño, 2006; Kalshoven et al., 2016). Indeed, although empirical evidence is scarce (Martin et al., 2016), research has predominantly suggested that leaders can *prevent* undesirable behaviors among their followers by forming high-quality LMX relationships with them (El Akremi et al., 2010; Liu et al., 2013; Jawahar et al., 2018). The central idea behind this preventive perspective is that high-quality LMX relationships obligate followers to positively reciprocate this relationship by reducing unethical behavior. Although the negative effect of positive reciprocity on pro-self unethical behavior in Study 1 certainly speaks to this idea, results of Study 2 demonstrate that positive reciprocity is positively associated with intended pro-self unethical behavior, suggesting moral licensing effects (cf. Sachdeva et al., 2009). Overall, therefore, our results imply that forming high-quality LMX relationships does not necessarily lead to a felt obligation among followers to reduce their pro-self unethical behavior.

Second, our findings have implications for the role of negative reciprocity in LMX relationships. Like positive reciprocity, negative reciprocity is part of the social exchange mechanisms that followers have at their disposal (Gouldner, 1960; Blau, 1964; Eisenberger et al., 2004). Although negative reciprocity is part of the LMX framework, it is not regularly used as such (Uhl-Bien and Maslyn, 2003). Instead, as mentioned earlier, research on LMX typically relies on the positive reciprocity route to argue for relationships with undesirable behavior (El Akremi et al., 2010; Liu et al., 2013; Jawahar et al., 2018). Moreover, although researchers have linked unethical behavior to positive reciprocity dispositions (Umphress et al., 2010; Bryant and Merritt, 2019), we know of no research that has considered the indirect effects of LMX on unethical behavior *through* positive reciprocity motives, not to mention negative reciprocity motives. This is an important shortcoming, given that leaders have limited resources to establish high-quality relationships with all their followers (Liden and Graen, 1980; Graen and Uhl-Bien, 1995; Henderson et al., 2009), meaning that negative reciprocity is very likely to result. Furthermore, our findings clearly indicate that negative reciprocity plays a crucial role in the relationship between LMX and unethical intentions. Specifically, developing low-quality LMX relationships may make followers feel sufficiently deprived that they have a need to negatively reciprocate this deprivation, which they can do by engaging in pro-self unethical behavior. Following up on our first theoretical implication, given that both high-quality (through positive reciprocity) and low-quality (through negative reciprocity) LMX relationships may elicit pro-self unethical behavior, forming LMX relations may not be useful for leaders to regulate follower unethical behavior.

Finally, our findings have implications for the further conceptualization of pro-leader relative to pro-self unethical behavior and how LMX relationships motivate it. Previous research has established that followers engage in pro-leader unethical behavior because they identify with their leader (Johnson and Umphress, 2018) and as a response to leader bottom-line mentality (Mesdaghinia et al., 2018). We add to this literature by consistently demonstrating that the high-quality LMX relationships that leaders develop with followers spark a necessity to positively reciprocate this relationship, which followers can do by engaging in pro-leader unethical behavior. Although previous studies have suggested that reciprocity considerations *moderate* the relationship between LMX and unethical behavior (e.g., Umphress et al., 2010; Bryant and Merritt, 2019), our social exchange theory embedded experimental approach demonstrates that positive and negative reciprocity *mediate* this relationship. These results not only indicate that pro-other unethical behavior is distinct from pro-self unethical behavior, as is frequently implied (e.g., Umphress et al., 2010; Umphress and Bingham, 2011; Johnson and Umphress, 2018), but also demonstrate that they operate through distinct mechanisms.

### Practical Implications

Our findings have meaningful implications for the promotion and prevention of unethical behavior through LMX relationships. Given its copious beneficial effects (Ilies et al., 2007; Dulebohn et al., 2012; Martin et al., 2016), differentiating among followers has become common managerial practice (Henderson et al., 2009). While we do not dispute that LMX relationships can be extremely useful and beneficial to management, our findings do suggest that LMX relationships may also have some qualities that limit their usefulness. Provided that both low- and high-quality LMX relationships motivate unethical behavior, albeit for different reasons, leaders are effectively motivating their followers to engage in unethical behavior through the LMX relationships that they establish – regardless of their quality. This Catch-22, where the beneficial effects of a management tool are associated with various harmful effects, is not unique to LMX relationships (e.g., goal-setting, Schweitzer et al., 2004; Ordóñez et al., 2009). One way of off-setting this perverse cycle, as previous research has suggested, is to employ followers high on moral identity (Aquino and Reed, 2002; Johnson and Umphress, 2018; Mesdaghinia et al., 2018), as this tends to reduce the effects of motivating mechanisms on unethical behavior. Given that moral identity is difficult to establish, however, it may be more efficient for a leader to emphasize moral awareness (Jordan, 2009). If leaders are able to create a moral awareness among their followers, they can reduce their intended unethical behaviors (Barsky, 2008). Leaders can potentially do so by employing an ethical leadership style that demotivates unethical conduct (Brown and Mitchell, 2010; Treviño et al., 2014). An alternative route for leaders to reduce unethical behavior is by increasing the likelihood and severity of punishment. If followers perceive that the punishment of unethical behavior outweighs its benefit or find that the behavior is not functional to satisfy their positive and negative reciprocity motives, their ethical inhibitions may be maintained (Chen et al., 2016; Vriend et al., 2016), causing them to refrain from engaging in such behavior (Becker, 1968; Mulder et al., 2015).

### Limitations and Future Research Directions

While our investigation has several strengths, it also has several limitations. Despite employing an experimental setup in a US sample and a time-lagged design in a Dutch context, there are several methodological and empirical limitations of note. First, both LMX and ethical decision-making are prone to cultural biases (Westerman et al., 2007; Rockstuhl et al., 2012). Although we found consistent evidence for our expectations in countries representing the Anglo and Nordic clusters (Ronen and Shenkar, 2013), the shape of our expected relationships may be different for other clusters. It seems feasible, for example, that collectivistic cultures are less open to negative reciprocity motives and self-serving unethical behavior than individualistic cultures, which may make the positive reciprocity and other-serving unethical behavior path more salient in these cultures. Second, similarly, our studies included followers from a wide range of organizations and industries. Although this attests to the external validity of our samples, it does not rule out that the shape of our expected relationships may be different for specific types of organizations or industries. Third, we used a single source, namely the follower, to gauge our focal variables. This implies that common method variance may bias (inflate) the relationships found across our studies. Given our experimental and time-split designs and that we found no evidence that the relatively small fraction of common method variance in Study 2 (5.24%) influenced the interrelationships and reliabilities of our focal variables, however, we do not think that common method variance is an issue.

Another methodological and empirical limitation of note is that we relied on self-report of unethical intentions, rather than other-reports of unethical behavior. Our argumentation to justify this is threefold. First, a follower’s unethical intentions are a cognitive representation that leaders are unable to tap into (cf. Janssen, 2000). Second, unethical acts violate important norms and can, in some cases, even be illegal. This means that followers are unlikely to reveal their unethical acts to others (Treviño and Brown, 2005), implying that it is difficult for leaders to assess the unethical intentions and behavior of their followers. Third, the average correlation between intention and behavior is relatively high (*r* = 0.47, as reported in a meta-analysis by Armitage and Conner, 2001); although unethical intentions do not perfectly capture unethical behavior, they should be a very strong predictor of it. Taken together, for the purpose of our study, we believe that measuring unethical intention is more appropriate than behavior and that this unethical intention sufficiently captures unethical behavior.

Both a conceptual and methodological limitation lies in the fact that LMX is a dynamic construct that continually changes (role theory; Graen, 1976) as a result of (reciprocal) interactions between leaders and followers (social exchange theory; Gouldner, 1960; Blau, 1964). This implies that LMX may be an endogenous construct (Antonakis et al., 2014) in which it is unclear whether LMX would have a causal effect on (pro-leader and pro-self) unethical behavior through (positive and negative) reciprocity, as we suggest, or whether this unethical behavior shapes the quality of the LMX relationship. This is further complicated by the fact that we rely on the argument that followers employ this unethical behavior to satisfy their reciprocity motive, likely intending to influence the quality of their LMX relationship. Although we have tried to relieve this limitation by employing an experimental setup in the first study and a time-split design in the field study in which participants were asked about their current LMX and their intended reciprocity motive and unethical behavior, this does not completely rule out alternative causal models.

A final conceptual limitation lies in our argumentation for the mediating role of negative reciprocity. We assume that negative reciprocity is caused by the fact that those in low-quality LMX relationships will feel deprived relative to those in high-quality LMX relationships. While there is ample evidence for this argument from an LMX differentiation perspective (Henderson et al., 2009), we do not empirically employ such a perspective, as we do not compare the LMX relationships between followers from the same leader. Furthermore, it could well be that followers have no need to establish social exchanges with their leaders and are relatively comfortable with relationships solely based on economic exchanges. In such cases, followers would not feel deprived, would not feel their economic exchange as a slight, and would have no need to engage in pro-self unethical behavior as a means of negative reciprocation. Hence, the preference of favoring a simple contract-based economic exchange relationship or wanting a higher-quality relationship could serve as an important moderator of the effects that we have explored throughout our studies.

## Conclusion

Scholars and practitioners have long assumed that leaders can prevent unethical behavior among their followers by establishing high-quality LMX relationships with them, which has become a popular means for leaders to manage their followers. Recent findings and the current investigation, however, have suggested and demonstrate that the story may be more nuanced, such that both low- and high-quality LMX relationships may motivate unethical behavior. Followers either engage in pro-leader unethical behavior to positively reciprocate high-quality relationships or pro-self unethical behavior to negatively reciprocate low-quality relationships. Regardless of their quality, therefore, LMX relationships motivate unethical behavior among followers. The only influence that the quality has, then, is who this unethical behavior is intended to benefit. In light of both its beneficial and harmful effects, theorists and practitioners should be wary of the effects of the LMX relationships: the dark side of relational leadership.

## Data Availability Statement

The datasets generated for this study are available on request to the corresponding author.

## Ethics Statement

All procedures performed in the studies were in accordance with ethical standards. All participants were informed about study procedures and voluntarily consented to participate. Study 1 was approved by the institutional research committee (University of Groningen Faculty of Economics and Business Ethical Committee, reference number #2013_52). The patients/participants provided their written informed consent to participate in this study.

## Author Contributions

TV and RS were involved in all steps of the research process. OJ and JJ contributed to the writing of the manuscript. All authors contributed to the article and approved the submitted version.

## Conflict of Interest

The authors declare that the research was conducted in the absence of any commercial or financial relationships that could be construed as a potential conflict of interest.

## Footnotes

1 The questionnaire initially contained two sets of six items. However, we left out three items across both sets because they referred to behaviors that were not unambiguously unethical. Furthermore, these items deviated from established unethical behavior scales (e.g., Umphress et al., 2010; Johnson and Umphress, 2018).
